# Diagnostic Challenges in a Patient With Systemic Lupus Erythematosus (SLE) and Anti-N-Methyl-D-Aspartate (NMDA) Receptor Encephalitis: A Case Report

**DOI:** 10.7759/cureus.93744

**Published:** 2025-10-02

**Authors:** Olga Vera-Lastra, Alfonso Carus-Sánchez, Ana L Peralta-Amaro, Abihai Lucas-Hernández, Emmanuel Rodríguez-Chávez

**Affiliations:** 1 Internal Medicine, Hospital de Especialidades Dr. Antonio Fraga Mouret, Instituto Mexicano del Seguro Social, Mexico, MEX; 2 Rheumatology, Hospital General de Teziutlán, Instituto Mexicano del Seguro Social (IMSS) BIENESTAR, Teziutlán, MEX; 3 Neurology, Hospital de Especialidades Dr. Antonio Fraga Mouret, Instituto Mexicano del Seguro Social, Mexico, MEX

**Keywords:** anti-glun1 antibodies, anti-glun2 antibodies, anti-n-methyl-d-aspartate receptor, autoimmune encephalitis, neuropsychiatric lupus, systemic lupus erythematosus

## Abstract

Anti-N-methyl-D-aspartate receptor encephalitis (NMDAR-E) is an autoimmune disorder characterized by psychiatric and neurological symptoms associated with the presence of anti-GluN1 antibodies in the cerebrospinal fluid (CSF). Neuropsychiatric systemic lupus erythematosus (NPSLE) encompasses 19 syndromes and remains a diagnostic challenge due to its heterogeneous clinical presentation and reliance on exclusion. While anti-GluN2 antibodies have been linked to systemic lupus erythematosus (SLE), the coexistence of NPSLE and NMDAR-E has been scarcely reported. Since the first case published in 2016, fewer than 15 patients have been documented worldwide, mostly as isolated case reports and only a few small series including patients with systemic autoimmune diseases such as SLE.

We report a 31-year-old woman with a one-year history of SLE who developed neuropsychiatric manifestations initially attributed to NPSLE. Despite treatment with high-dose corticosteroids, cyclophosphamide, and antipsychotics, she experienced recurrent psychotic episodes with poor response. Brain magnetic resonance imaging (MRI) showed nonspecific hyperintense lesions, and CSF analysis revealed positive anti-GluN1 antibodies, confirming the diagnosis of NMDAR-E. The patient improved after intravenous immunoglobulin and plasmapheresis, and she remained stable under maintenance therapy with azathioprine and rituximab.

This case illustrates the diagnostic complexity of neuropsychiatric manifestations in SLE, where overlapping autoimmunity may conceal the presence of NMDAR-E. Clinicians should maintain a high index of suspicion for autoimmune encephalitis in SLE patients with refractory neuropsychiatric symptoms, as timely recognition and treatment can significantly improve outcomes.

## Introduction

Anti-N-methyl-D-aspartate receptor encephalitis (NMDAR-E) is an autoimmune disorder characterized by IgG antibodies against the GluN1 subunit of the NMDA receptor in cerebrospinal fluid (CSF), leading to a wide range of neuropsychiatric symptoms, including acute psychosis, seizures, movement disorders, and cognitive impairment [1,2]. Since its first description in 2007, NMDAR-E has become the most commonly identified subtype of autoimmune encephalitis (AIE), with a prevalence approaching that of viral encephalitis in specialized centers [3].

Neuropsychiatric systemic lupus erythematosus (NPSLE) includes 19 different syndromes, from mild cognitive problems to severe psychosis or cerebrovascular issues [4,5]. Although it is primarily a diagnosis of exclusion, specific autoantibodies, such as anti-ribosomal P and anti-NR2 (GluN2) antibodies, are involved in its development. These antibodies can cross-react with neuronal NMDA receptors, leading to excitotoxicity and neuronal damage [6-8].

While both NMDAR-E and NPSLE share overlapping clinical features, they differ in their immunological targets: NMDAR-E is associated with anti-GluN1 antibodies, whereas NPSLE is more commonly linked to anti-GluN2 antibodies [9]. This difference is clinically significant because patients with NMDAR-E often respond to immunotherapies such as intravenous immunoglobulins (IVIg), plasma exchange, and rituximab. Meanwhile, NPSLE is typically treated with high-dose corticosteroids and standard immunosuppressants [1,3,10].

Reports describing the coexistence of systemic lupus erythematosus (SLE) and NMDAR-E are rare, with only isolated cases and small series published to date [11-14]. Distinguishing between these two conditions is essential for clinical decision-making, particularly in SLE patients with refractory neuropsychiatric symptoms. Here, we present the case of a young woman with SLE who experienced recurrent psychosis, initially attributed to neuropsychiatric lupus, but ultimately diagnosed with NMDAR-E after anti-GluN1 antibodies were identified in the CSF [11]

## Case presentation

**Figure 1:**
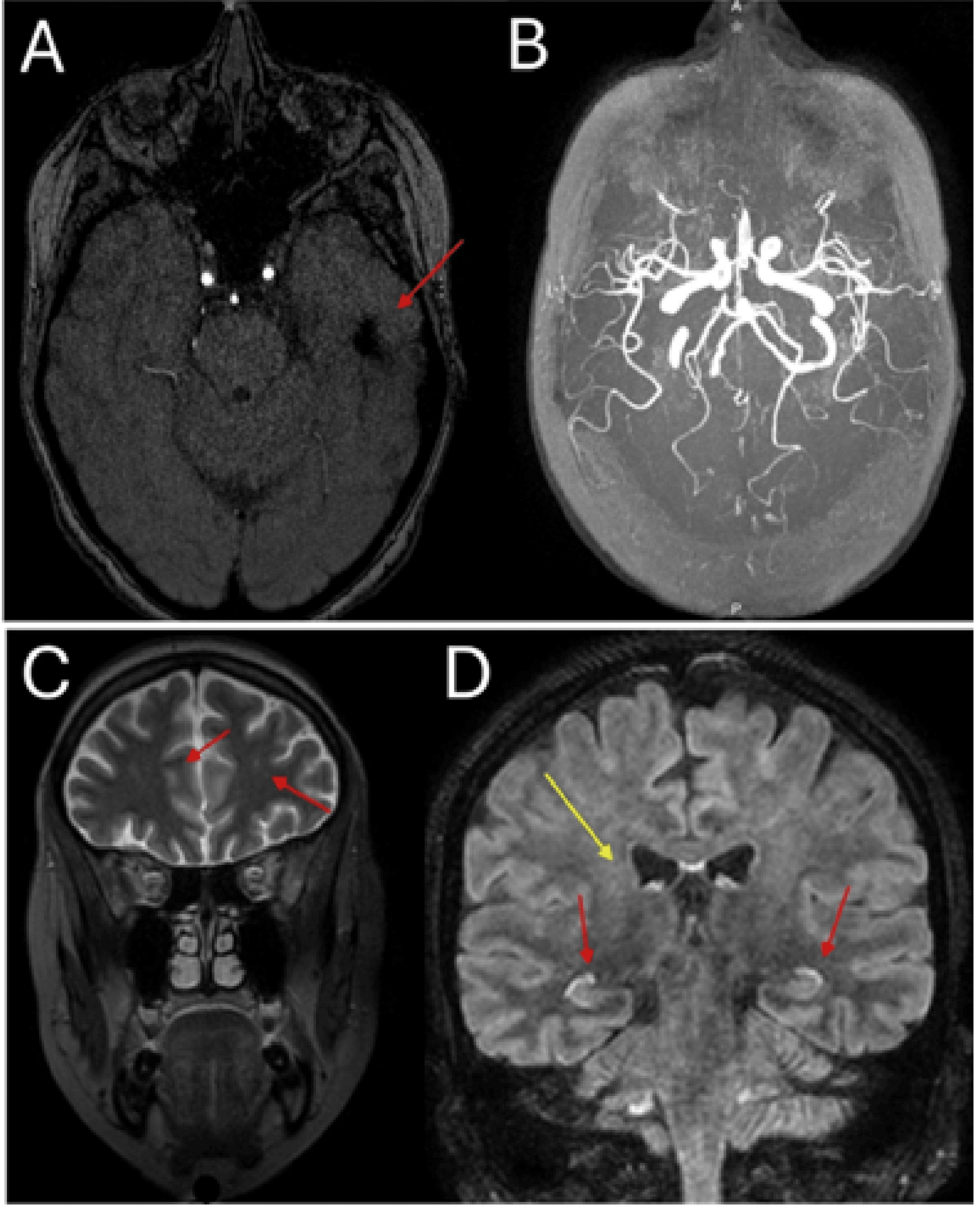
Brain imaging findings in a patient with systemic lupus erythematosus and anti-NMDAR encephalitis. (A) Cranial CT angiography showing a hypodense left frontotemporal lesion consistent with an old infarct. (B) No radiological evidence of cerebral vasculitis. (C) Brain MRI (T2 sequence) showing nonspecific hyperintense lesions in the pyramidal tract and prefrontal regions (arrows). (D) Brain MRI (T1 sequence) showing hippocampal hyperintensity (red arrows) and pyramidal tract hyperintensity (yellow arrows). NMDAR, N-methyl-D-aspartate receptor

In February 2022, a 31-year-old woman was diagnosed with SLE based on cutaneous and articular manifestations, positive antinuclear antibodies (ANA), and anti-dsDNA antibodies. Initial treatment with hydroxychloroquine and prednisone resulted in a good clinical response. One year later, in January 2023, she developed recurrent cutaneous activity and mild proteinuria. In this context, mycophenolate mofetil was added to her regimen. In February 2023, the patient experienced her first severe neuropsychiatric episode characterized by disorganized thinking, persecutory delusions, auditory hallucinations, language disturbances, decreased need for sleep, and psychomotor agitation. At that time, CSF analysis and electroencephalogram were unremarkable, while cranial CT angiography revealed an old left frontotemporal infarct without signs of vasculitis (Figure 1).

Laboratory tests revealed hypocomplementemia and elevated anti-dsDNA antibodies, findings consistent with NPSLE (Table 1).

**Table 1:** Serum laboratory results and cerebrospinal fluid analysis in a patient with autoimmune encephalitis and systemic lupus erythematosus. Reference ranges are provided for quantitative analytes. Qualitative assays (CSF/urine sediment, anti-GluN1 IgG, anti-Sm) are reported categorically; the reference value is *Negative*, as no numerical range applies. Positivity reflects reactivity above the manufacturer’s validated threshold for the specific assay used.

Test	Result	Normal values
Cerebrospinal fluid (CSF)		
Glucose	66 mg/dL	45-75 mg/dL
Lactate dehydrogenase (LDH)	20 U/L	0-45 U/L
Microproteins	19.8 mg/dL	<20 mg/dL
Leukocytes	0/mm³	0-5/mm³
Sediment	Negative	Negative
Anti-GluN1 IgG antibodies	Positive	Negative
Immunological studies		
Antinuclear antibodies (ANAs)	1:160 homogeneous	<1:80
Complement C3	60 mg/dL	90-180 mg/dL
Complement C4	13 mg/dL	10-40 mg/dL
Anti-DNA (double-stranded)	54 U/mL	<20 U/mL
Anti-Smith	Negative	Negative
Serum Studies		
Hemoglobin	11.2 g/dL	12-16 g/dL (female)
Leukocytes	5.5 x 10³/µL	4.0-10.0 x 10³/µL
Lymphocytes	1.06 x 10³/µL	1.0-4.0 x 10³/µL
Platelets	397 x 10³/µL	150-450 x 10³/µL
Creatinine	0.59 mg/dL	0.6-1.2 mg/dL
Glucose	68 mg/dL	70-100 mg/dL (fasting)

**Figure 2:**
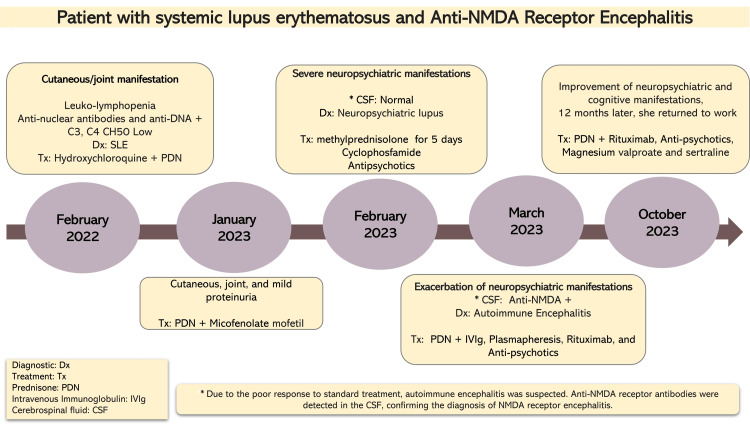
Timeline of the patient’s clinical course. The figure summarizes the chronological sequence of clinical manifestations, diagnostic evaluations, and therapeutic interventions. Image credit: All authors. CSF, cerebrospinal fluid; SLE, systemic lupus erythematosus; PDN, prednisolone; NMDA, N-methyl-D-aspartate

She was hospitalized and treated with methylprednisolone pulses (1 g/day for five days), followed by a single intravenous pulse of cyclophosphamide (750 mg/m²), together with atypical antipsychotics. This approach led to only partial improvement, suggesting an incomplete response. In March 2023, she suffered a relapse with severe psychosis refractory to high-dose antipsychotics, behavioral modulators, and benzodiazepines. The brain MRI revealed nonspecific hyperintense lesions in the pyramidal tract, hippocampus, prefrontal gyrus, and substantia nigra (Figure 1). Given the atypical course and poor treatment response, CSF was tested for anti-GluN1 IgG antibodies, which were positive, confirming a diagnosis of anti-NMDAR-E. Infectious and neoplastic causes were excluded. Following this diagnosis, the patient was subsequently treated with IVIg, followed by plasmapheresis, which resulted in significant clinical improvement. Maintenance therapy with azathioprine and rituximab was subsequently initiated, resulting in sustained clinical stability. By October 2023, the patient remained stable under follow-up, receiving prednisone, rituximab, antipsychotics, magnesium valproate, and sertraline. She was able to return to work and daily activities, underscoring the importance of timely recognition and tailored therapy in cases where overlapping autoimmunity complicates the clinical picture. The overall clinical course is summarized in a timeline (Figure 2).

## Discussion

This patient initially met criteria for NPSLE and later developed features consistent with NMDAR-E. Neuropsychiatric involvement in SLE remains a significant diagnostic challenge and continues to be the second leading cause of morbidity and mortality in these patients [4,5]. From a pathophysiological perspective, neurological manifestations may result from vascular injury mediated by cerebral microangiopathy or antibody-mediated neuronal dysfunction [5,6].

Multiple autoantibodies have been linked to NPSLE, especially anti-ribosomal P and anti-NR2 antibodies, the latter targeting a subunit of the NMDA receptor [6-8]. These antibodies can cross-react with neuronal NMDA receptors, leading to excitotoxicity, alterations in synaptic transmission, and neuronal cell death. NMDAR-E is an immune-mediated disorder marked by IgG antibodies against the GluN1 subunit of the NMDA receptor in CSF, which directly interfere with receptor function and neuronal signaling [1,2,9]. Although their immunological targets differ, both conditions involve the NMDA receptor pathway, providing a biological explanation for their overlapping clinical features. Since the discovery of anti-GluN1 antibodies, the diagnostic approach for AIE has undergone considerable changes. The Graus criteria published in 2016 specify that the presence of GluN1 IgG antibodies in CSF, along with a subacute (<3 months) onset of psychiatric or neurological symptoms and the exclusion of infectious causes, is enough for diagnosis [3]. Notably, up to 59% of patients with AIE exhibit psychiatric symptoms, often leading to misdiagnosis as primary psychosis [10].

In our patient, the initial presentation of psychosis, along with hypocomplementemia and elevated anti-dsDNA antibodies, supported a diagnosis of NPSLE. Corticosteroid pulses and cyclophosphamide are standard treatments for this condition, initially resulting in partial improvement. However, the subsequent relapse, poor response to antipsychotics, and detection of anti-GluN1 antibodies in CSF confirmed NMDAR-E. This case highlights the need for clinicians to remain vigilant when neuropsychiatric symptoms persist despite conventional immunosuppression. Management strategies for NPSLE and NMDAR-E vary significantly. NPSLE is typically treated with high-dose corticosteroids and conventional immunosuppressants such as cyclophosphamide or mycophenolate [5,11].

In contrast, first-line therapy for NMDAR-E includes IVIg or plasma exchange, often combined with corticosteroids, while second-line options usually involve rituximab or cyclophosphamide [9,10,12]. Our patient achieved clinical remission only after IVIg and plasmapheresis, and she remained stable on azathioprine and rituximab, demonstrating the importance of tailoring therapy to the underlying pathogenic mechanism when overlapping autoimmunity is suspected. Although anti-GluN1 antibody testing in cerebrospinal fluid is not routinely recommended for patients with SLE, its consideration may be warranted in atypical or treatment-refractory neuropsychiatric cases. In these situations, detecting anti-GluN1 antibodies can be crucial for differentiating NMDAR encephalitis from NPSLE and guiding specific therapy [1,3,9,10]. In our patient, CSF antibody testing was performed only after the relapse of neuropsychiatric symptoms, underscoring the diagnostic challenge in differentiating NPSLE from NMDAR-E. This delay reflects the difficulty clinicians may face when persistent psychiatric manifestations are initially attributed to lupus activity rather than AIE [2,3,10].

The recognition of AIE as a distinct clinical condition has revolutionized the field of neuroimmunology. Over the past 15 years, its prevalence has approached that of viral encephalitis in specialized centers [3]. While most cases are idiopathic or paraneoplastic, there is a growing number of reports linking it to systemic autoimmune diseases. DeGiorgio et al. demonstrated that anti-dsDNA antibodies in SLE can cross-react with NMDA receptors, leading to neuronal damage [8]. Meanwhile, Hirohata and Tanaka confirmed that antibodies against the NR2 subunit are found in up to 61% of NPSLE patients, compared to just 6% with antibodies against the entire receptor complex [9].

The first case of SLE associated with NMDAR-E was reported by Wu et al. [11]. Since then, isolated cases and small series have been reported, including recent publications that emphasize the diagnostic challenges in SLE patients with neuropsychiatric symptoms [12-14]. Our report contributes to this growing evidence, supporting the concept of a *kaleidoscope of autoimmunity*, where systemic and organ-specific autoimmune diseases can coexist and overlap [15]. From a clinical perspective, this case underscores the importance of maintaining a broad differential diagnosis when evaluating neuropsychiatric symptoms in patients with SLE. Although NPSLE is frequently the first consideration, persistence of severe symptoms despite conventional immunosuppression should prompt clinicians to consider alternative causes, including AIE. Recognizing the possibility of overlapping autoimmunity and testing for anti-GluN1 antibodies in selected cases may allow timely and targeted therapy, ultimately improving outcomes. This case, therefore, provides a practical framework for approaching similar diagnostic dilemmas in daily practice.

## Conclusions

This case illustrates the complexity of neuropsychiatric manifestations in SLE and the challenge in differentiating them from NMDAR-E. Although both conditions share overlapping clinical features, they differ in immunological targets and therapeutic responses. Beyond the individual case, this report underscores the broader clinical relevance of considering AIE in the differential diagnosis of refractory or atypical NPSLE. It supports the incorporation of CSF antibody testing into future diagnostic strategies. Early recognition and tailored treatment may improve patient outcomes.
